# The Tissue Distribution and Urinary Excretion Study of Gallic Acid and Protocatechuic Acid after Oral Administration of *Polygonum Capitatum* Extract in Rats

**DOI:** 10.3390/molecules21040399

**Published:** 2016-03-24

**Authors:** Feng-Wei Ma, Qing-Fang Deng, Xin Zhou, Xiao-Jian Gong, Yang Zhao, Hua-Guo Chen, Chao Zhao

**Affiliations:** 1 Key Laboratory for Information System of Mountainous Areas and Protection of Ecological Environment, Guizhou Normal University, Guiyang 550001, China; mfw200422501212@163.com (F.-W.M.); dqf_2012@126.com (Q.-F.D.); gongxiaojian1@163.com (X.-J.G.); bestyangzhao@gmail.com (Y.Z.); huaguochen81@gmail.com (H.-G.C.); chaozhao@126.com (C.Z.); 2 Guizhou Engineering Laboratory for Quality Control & Evaluation Technology of Medicine, Guizhou Normal University, 116 Baoshan North Road, Guiyang 550001, China; 3 The Research Center for Quality Control of Natural Medicine, Guizhou Normal University, 116 Baoshan North Road, Guiyang 550001, China

**Keywords:** distribution, excretion, *Polygonum capitatum* extract, gallic acid, protocatechuic acid

## Abstract

In the present study, we investigated the tissue distribution and urinary excretion of gallic acid (GA) and protocatechuic acid (PCA) after rat oral administration of aqueous extract of *Polygonum capitatum* (*P. capitatum*, named Herba Polygoni Capitati in China). An UHPLC-MS/MS analytical method was developed and adopted for quantification of GA and PCA in different tissue homogenate and urine samples. Interestingly, we found that GA and PCA showed a relatively targeted distribution in kidney tissue after dosing 60 mg/kg *P. capitatum* extract (equivalent to 12 mg/kg of GA and 0.9 mg/kg of PCA). The concentrations of GA and PCA in the kidney tissue reached 1218.62 ng/g and 43.98 ng/g, respectively, at one hour after oral administration. The results helped explain the empirical use of *P. capitatum* for kidney diseases in folk medicine. Further studies on urinary excretion of *P. capitatum* extract indicated that GA and PCA followed a concentrated elimination over a 4-h period. The predominant metabolites were putatively identified to be 4-methylgallic acid (4-OMeGA) and 4-methylprotocatechuic acid (4-OMePCA) by analyzing their precursor ions and characteristic fragment ions using tandem mass spectrometry. However, the amount of unchanged GA and PCA that survived the metabolism were about 14.60% and 15.72% of the total intake, respectively, which is reported for the first time in this study.

## 1. Introduction

Herba Polygoni Capitati, the aerial part or whole plant of *Polygonum capitatum* Buch.-Ham. *ex* D. Don, has been used as a traditional Miao-nationality herb for the treatment of urinary tract infections and pyelonephritis [1,2]. *Polygonum capitatum* (*P. capitatum*) is a perennial plant belonging to the Polygonaceae family and mainly distributed in India, Nepal and the southwest provinces of China [3]. Phytochemical analysis of *P. capitatum* showed that main chemical components are polyphenols [3,4,5]. Modern pharmacological investigations have indicated that aqueous extract of *P. capitatum* has antioxidant [6], antibacterial [7], and anti-inflammatory activities [8].

The *in vivo* distribution study of a candidate is indispensable in drug research as it demonstrates its pharmacokinetic features [9]. Investigation of drug distribution in the whole-body, which provides insight into the drug’s accumulation and metabolism in a particular tissue, is especially useful [10]. For herbal medicines, however, due to the complexity of their chemical constituents, a rational approach has been the use of one or several compounds in generous amounts as markers to establish these herbal medicines’ tissue distribution profiles [11,12,13,14]. Gallic acid (GA) and protocatechuic acid (PCA) are abundant in *P. capitatum* and GA was also selected as the target marker for quality control of *P. capitatum* in the Chinese Pharmacopoeia [1]. GA and PCA are well-known polyphenol compounds that exist in the daily diet and many herbal medicines, and have long been demonstrated to have remarkable anti-radical [15], anti-inflammatory [16], antitumor [17,18], antioxidant [19] activities and hepatoprotective effects [20,21]. Recently, GA was also reported to have neuron-protective [22] and renal-protective effects [23,24]. It is generally accepted that the bioavailability of polyphenols is relatively poor, although GA shows a quick absorption [25]. After digestion, metabolized polyphenols can lose their original properties or even may exert different activities [24,26]. Given the complexity of the typical herbal medicine matrix, the intake of polyphenol-rich products is not directly linked to the profiles observed after an acute intake of these pure polyphenol compounds [27,28,29]. The tissue distribution and urinary excretion study of GA and PCA after oral administration of aqueous extract of *P. capitatum* to rats is a meaningful way of illustrating its empirical use in folk medicine, so GA and PCA were picked as the markers for our *P. capitatum* tissue distribution study.

Recently, several new techniques have been adopted for the analysis of drug distribution by mass spectrometry [30,31]. With improved resolving power for discriminating mass/charge (*m/z*) ratios, the application of MS to the identification and quantification of chemical compounds has become much more extensive and comprehensive [32,33]. In the present study, an UHPLC-MS/MS analytical method for GA and PCA analysis was developed and validated, and thereafter, the tissue distribution and urinary excretion of GA and PCA after oral administration of *P. capitatum* extract were investigated. The predominant metabolites in rat urine were also putatively identified by analyzing their precursor ions and characteristic fragment ions by tandem mass spectrometry. Some noteworthy findings were obtained through the tissue distribution and urinary excretion studies.

## 2. Results and Discussion

### 2.1. Method Development and Validation

#### 2.1.1. Selectivity and Matrix Effects

**Figure 1:**
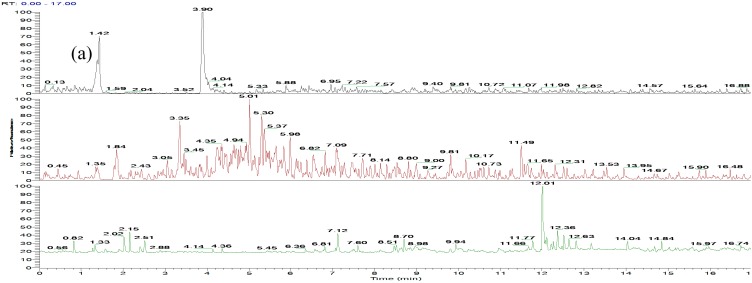

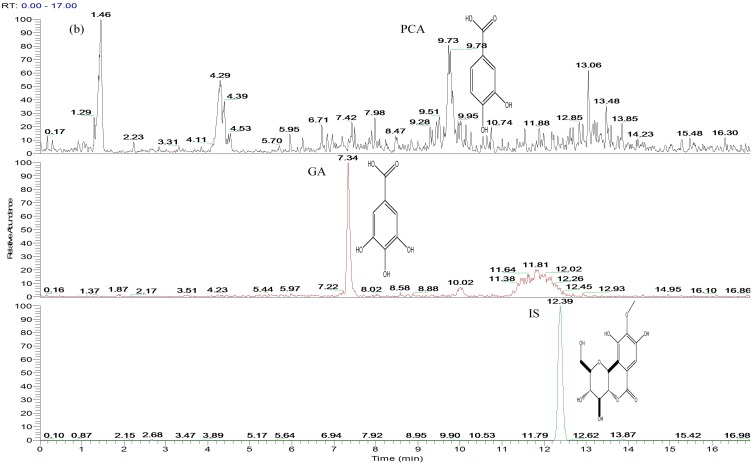

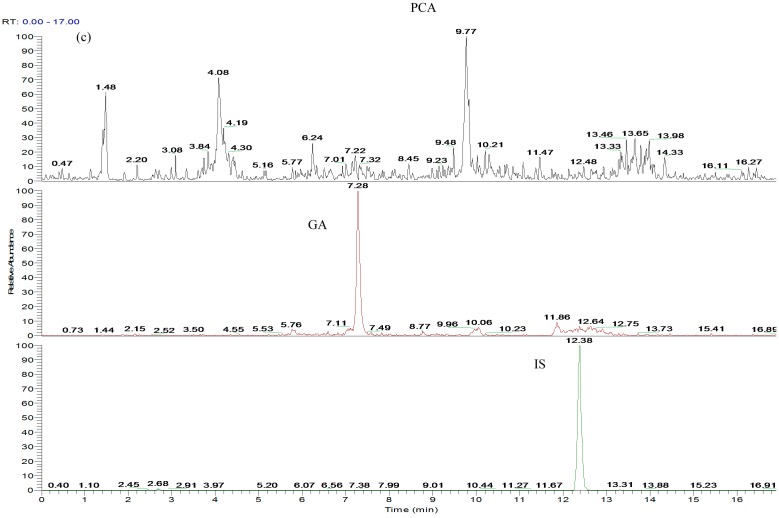
Representative MRM chromatograms of GA, PCA and IS (bergenin) in rat kidney: (**a**) blank kidney homogenate; (**b**) blank kidney homogenate spiked with GA, PCA and IS at LLOQ concentration of 30.00, 9.95 and 10.01 ng/mL, respectively; (**c**) kidney homogenate at 60 min after oral administration of 60 mg/kg *P. capitatum* extract (184.68 ng/mL for GA and 11.82 ng/mL for PCA).

All analytes, including bergenin (the internal standard, IS), were monitored under negative ionization conditions and quantified in multiple reactions monitoring (MRM) mode. Under the UHPLC-MS/MS conditions, there were no interfering peaks at the elution times of GA, PCA and the IS. An example of the analysis of a kidney homogenate is shown in Figure 1, where the typical MRM chromatograms of blank kidney tissue (a), spiked kidney tissue containing GA, PCA, and IS at their LLOQ concentration (b), and kidney tissue collected at 60 min after oral administration of *P. capitatum* extract (c) were well separated and the peak shapes were satisfactory. The matrix effect of different tissue homogenate was negative for the analysis of GA and PCA in different tissues. The developed UHPLC-MS/MS method was also successfully adopted for analysis GA and PCA in urine samples. A chromatogram of a urine sample is presented in Supplementary Materials Figure S1.

#### 2.1.2. Linearity and LLOQ

Calibration curves plots of analyte-to-IS peak-area ratio (*y*) against concentration (*x*) were constructed using 1/*x*^2^ weighed least-squares linear regression [34]. The equations for the plots, the correlation coefficients, and the linear ranges for GA and PCA in different tissue and urine are listed in Table 1. All calibration curves showed good linearities (γ^2^ > 0.99) within the test ranges. The lower limit of quantification (LLOQ) was defined as the lowest drug concentration that can be determined with RE and *RSD* < 20%. LLOQs for all bio-samples are also shown in Table 1.

#### 2.1.3. Precision, Accuracy and Extraction Recovery Tests

The intra- and inter-day precision and accuracy of GA and PCA were assessed by analyzing quality control (QC) samples at three concentrations in six duplicates. The accuracy was within ±15% (20% for LLOQ), and the intra- and inter-day precisions were not to exceed ±15% (20% for LLOQ). The absolute recoveries of GA and PCA in different tissue homogenate and urine samples were high and consistent for the analysis. The data of precision, accuracy and extraction recovery tests were shown in Supplementary Materials Table S1. The results demonstrated that the values met the requirements of the current Chinese Pharmacopoeia and the method was accurate and precise for GA and PCA analysis in complex matrixes.

#### 2.1.4. Stability

The stability tests were designed to cover the expected conditions that real samples may experience. QC samples at two concentrations (1700 ng/mL for GA and 85 ng/mL for PCA, respectively) were used. Room temperature stability (4 h), post-preparative stability (4 °C for 24 h), freeze-thaw stability after three cycles (−20 °C to room temperature as one cycle) and long term stability (−20 °C for 7 days) were tested. The results are presented in Supplementary Materials Table S2, which indicated that the two analytes were stable under routine laboratory conditions.

### 2.2. Tissue Distribution Study

The developed UHPLC-MS/MS method was successfully applied to the determination of GA and PCA in tissues of rats after oral administration of *P. capitatum* extract. The rat heart, liver, spleen, lung, kidney, and brain tissues were removed 60 min after dosing. The tissue concentrations of the two analytes were determined using the developed UHPLC-MS/MS method.

GA and PCA were mainly distributed in kidney tissue, reaching 1218.62 ng/g and 43.98 ng/g, respectively, after oral administration of *P. capitatum* extract at a single dose equivalent to 12 mg/kg for GA and 0.9 mg/kg PCA. The lung tissue was found to be the second preference for GA and PCA distribution after oral administration of *P. capitatum* extract, and the content was 258.08 ng/g for GA and 19.48 ng/g for PCA, respectively. Neither GA nor PCA could be found in brain tissue, indicating the difficulty in crossing the “blood-brain” barrier (the data is shown in Supplementary Materials Table S3). 

**Figure 2:**
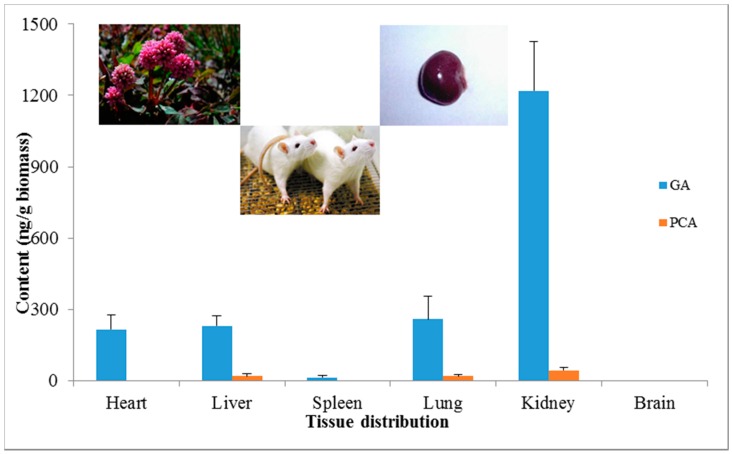
Tissue distributions of GA and PCA in various rat organs after oral administration of 60 mg/kg *P. capitatum* extract.

Generally, studies on GA are mainly focused on its anti-oxidant, anti-inflammatory and anti-carcinogenic effects [22,35,36]. As for clinical use, GA is commonly employed as a food additive or anti-obesity agent [37,38]. Other studies have implied that the intake of GA might help diabetes and cardiovascular diseases [39,40]. However, there was little information about the renal effects of GA until Peng and co-workers reported the positive therapeutic effect of GA in the treatment of chronic kidney disease [23]. Herein, we found that GA and PCA were mainly distributed in kidney tissue after rat oral administration of *P. capitatum* extract (Figure 2), which presents a positive correspondence with the usage of *P. capitatum* in treating renal diseases by the traditional Miao-nationality.

### 2.3. Excretion Study

Shahrzad and co-workers have previously proposed an HPLC method to determine GA and its metabolites in human urine [41]. In this study, we used an UHPLC-MS/MS method for GA and PCA determination, which was simple, rapid and selective. The LLOQ was 30 ng/mL and there was no need for a hydrolysis step prior to analysis. Our intuition about GA is that GA undergoes intensive metabolic bio-transformations in mammals, and is then converted into a number of metabolites via methylation and/or conjugations in the mammalian gut and liver [41,42,43,44,45].

**Figure 3:**
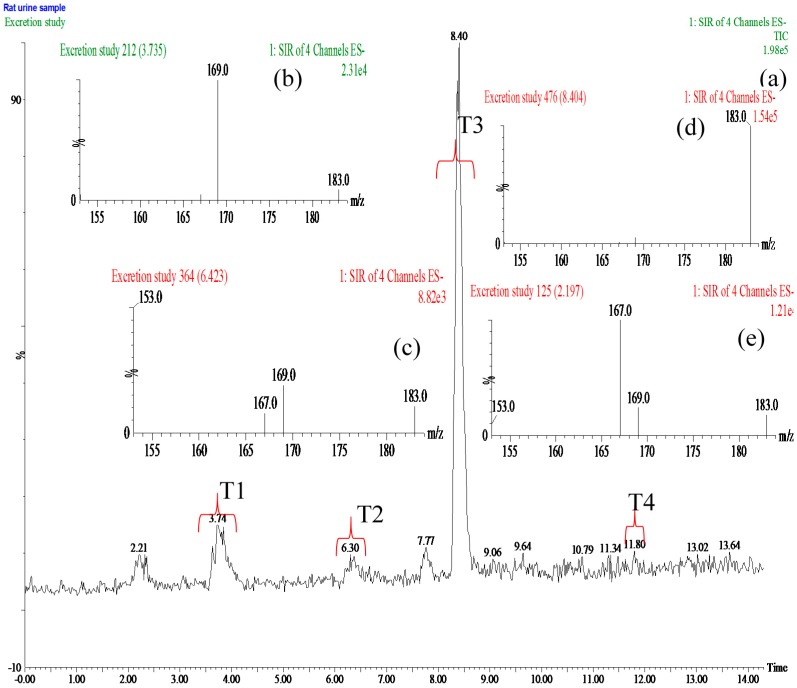
(**a**) Total ion chromatogram (TIC) of rat urine sample with selective ion monitoring (SIM) four ions at *m/z* 153, 167,169 and 183 in negative ion mode after oral administration of 60 mg/kg *Polygonum capitatum* extract; (**b**–**e**) Mass spectra of GA, PCA, 4-OMeGA and 4-OMePCA obtained from the period of T1, T2, T3 and T4, respectively.

In the present study, we investigated the excretion of GA and PCA after oral administration of *P. capitatum* extract. Firstly, selective ion monitoring (SIM) mode was used for mass spectrometric qualitative analysis of GA and PCA metabolites in rat urine after oral administration of 60 mg/kg *P. capitatum* extract (equivalent to 12 mg/kg for GA and 0.9 mg/kg PCA). 4-Methylgallic acid (4-OMeGA) and 4-methylprotocatechuic acid (4-OMePCA) were found in rat urine (Figure 3). Further structural identification of 4-OMeGA was putatively confirmed by its representative characteristic fragment ions under collision-induced dissociation using tandem mass spectrometry. (Data is shown in Supplementary Materials Figure S2).

**Figure 4:**
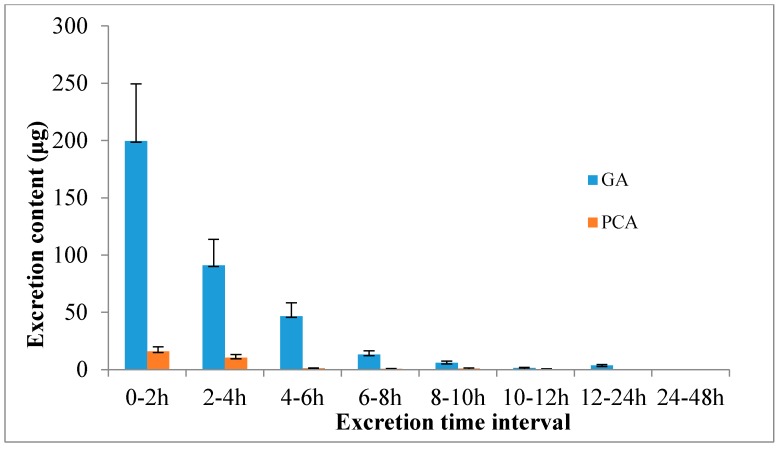
Excretion profiles of GA and PCA in rat after oral administration of 60 mg/kg *P. capitatum* extract.

However, the mean amount of unchanged GA and PCA were 361.44 μg and 29.55 μg in urine collected after 0 h to 48 h, respectively. The unmetabolized GA and PCA amounts were measured at 14.60% of the GA intake and 15.72% of the PCA intake, respectively, after oral administration of *P. capitatum* extract, which is first being reported in this study (Table 2). Another noteworthy finding was that most of the GA and PCA were excreted in the first 4 h after oral administration of *P. capitatum* extract, which accounted for more than 80% of the total excreted amount (Figure 4).

## 3. Experimental Section

### 3.1. Materials and Reagents

*Polygonum capitatum* Buch.-Ham. *ex* D. Don was supplied by Guizhou Warmen Pharmaceutical Co., Ltd. (Guizhou, China) and identified by professor Deyuan Chen. A voucher specimen was deposited at the Research Center for Quality Control of Natural Medicine (Guizhou Normal University, Guizhou, China). Authentic standards of GA (110831-200803) and bergenin (1532-200202) were purchased from the National Institute for the Control of Biological and Pharmaceutical Products of China (Beijing, China). PCA (0101-201011) was purchased from Guizhou Dida Biological Technology Co., Ltd. (Guizhou, China). HPLC grade acetonitrile and methanol were obtained from Tedia Co. Inc. (Fairfield, OH, USA). Formic acid was of MS grade (Roe Scientific Inc., Cincinnati, OH, USA). Super purified water from Robust Food & Beverage Co. Ltd. (Hong Kong, China) was used for all preparations. All other solvents used in the present study were of analytical grade and commercially available.

### 3.2. Experimental Animals

Pathogen-free adult male Sprague-Dawley (SD) rats weighing 250 ± 30 g were purchased from Changsha Tianqin Bio-technology Co., Ltd. (Hunan, China, Certificate No. SCXK 2009-0012). All rats were acclimated for at least a week before the study. Upon arrival, animals were randomized and housed at three per cage under controlled environmental conditions (24 ± 1 °C and 12/12 h light/dark cycle) with free access to standard chow and water. The rats were fasted overnight but were supplied with water *ad libitum* before the experiments. The protocols for the animal studies were approved by the Institute of Laboratory Animal Resources of Guizhou Normal University (Guizhou, China).

### 3.3. Analytical Method

The UHPLC-MS/MS system consisted of an Accela 1250 UHPLC system equipped with a TSQ quantum ultra-triple-quadrupole mass spectrometer (Thermo Fisher Scientific Inc., Waltham, MA, USA). Chromatographic separations were performed on a Phenomenex Kinetex XB-C18 column (150 × 2.1 mm, 1.7 μm). A guard column (Raptor C18 EXP Guard Column Cartridge, 5 × 2.1 mm, 2.7 μm) was used to provide protection of the costly analytical columns for analysis these complex matrix samples. The mobile phases consisted of solvent A: acetonitrile-formic acid (0.1%) and solvent B: water–formic acid (0.1%). The isocratic elution program was as follows: 0–3.5 min, 2% A; 3.5–12.5 min, 10% A; 12.5–17.0 min, 2% A. The column temperature was maintained at 25 C. The flow rate was 200 μL/min and the injection volume was 10 μL. After each injection, a needle wash with methanol was performed. Mass spectrometric analyses were performed on a TSQ quantum ultra-triple-quadrupole mass spectrometer (Thermo Fisher Scientific Inc.) equipped with an electro-spray ionization (ESI) interface in negative mode. All analytes, including the IS, were monitored under negative ionization conditions and quantified in multiple reactions monitoring (MRM) mode with transitions of *m/z* 169.012 → 125.042 for GA, *m/z* 153.114 → 109.201 for PCA, and *m/z* 326.930 → 192.017 for IS. Other parameters of the mass spectrometer were as follows: sheath gas flow rate, 40 (arbitrary units); auxiliary gas flow rate, 10 (arbitrary units); spray voltage, 2500 V; vaporizer temperature, 400 °C; capillary temperature, 400 °C. Helium was used as the collision gas for collision-induced dissociation (CID). The values of tube lens offset, helium collision gas pressure and collision energy for each parent ion-product ion transition were displayed in Supplementary Materials Table S4.

### 3.4. Preparation of P. capitatum Extract

For the preparation of *P. capitatum* extract, 1 kg of the dried powder (60 mesh) was accurately weighed into a 5 L glass round-bottom flask and extracted with 3 L of water under reflux for 1.5 h. The extract was centrifuged at 3000 rpm for 10 min and filtered through filter paper, and then the filtrate was evaporated to dryness for intragastric administration. Before oral administration of *P. capitatum* extract, 6 mg/mL (equivalent to 0.4 mg/mL of GA and 0.03 mg/mL of PCA) of *P. capitatum* extract was prepared by suspending the *P. capitatum* extract in water. When conducting the experiment, every rat doses proper volume of the *P. capitatum* extract suspension according to their body weight (1 mL/100 g body weight).

### 3.5. Method Validation

The developed UHPLC-MS/MS bioanalytical method was validated according to the accepted FDA Guidance for Industry [34], including selectivity, matrix effect, calibration curves, lower limit of detection and quantification, extraction recovery, precision, accuracy and stability. The endogenous compounds should not interference the analysis of GA and PCA at the retention time. The matrix effects of GA and PCA was measured by using quality control samples at three different concentration levels. Extraction recoveries were determined by comparing the peak areas of GA and PCA pre-processing and post-processing procedures.

### 3.6. Distribution Studies

After a single oral dose of 60 mg/kg *P. capitatum* extract to each rat, rats were killed by decapitation after 60 min. The heart, liver, spleen, lung, kidney and brain tissue were removed after perfusion with normal saline solution (0.9%) to eliminate blood contamination respectively. These dissected organs were weighed and then homogenized in saline solution (fivefold tissue weight) using a model 985370-395 variable speed tissue tearor (BioSpec Products Inc., Bartlesville, OK, USA). The tissue homogenates obtained were centrifuged at 5000 rpm for 15 min and the supernatants were stored at −20 °C until analysis.

A 100 μL aliquot of tissue homogenate was transferred into a 1.5 mL Eppendorf tube (EP tube), and 10 μL IS solution (0.1 μg/mL) and 400 μL 2% formic acid acetonitrile were individually added. The resulting solution was thoroughly vortex-mixed for 60 s and then centrifuged at 12000 rpm for 10 min at 4 °C. Subsequently, the supernatant was transferred into a clean 1.5 mL EP tube and evaporated to dryness under a nitrogen stream at 40 °C. The residue was re-dissolved in 100 μL of 0.1% formic acid aqueous solution and centrifuged. A 10 μL aliquot was injected into UHPLC-MS/MS for analysis.

### 3.7. Metabolism and Excretion Studies

Three rats were picked for metabolism study of GA and PCA after oral dose *P. capitatum* extract. After dosing 60 mg/kg *P. capitatum* extract (equivalent to 12 mg/kg for GA and 0.9 mg/kg PCA), each rat was housed individually in metabolic cages equipped with a urine and feces separator. Urine samples was collected after 60 min and stored immediately at −20 °C. Urine sample was prepared using liquid-liquid extraction, according to the following methods: 400 μL of ethyl acetate was added to 100 μL urine sample, and then was vortexed for 60 s before centrifugation at 12,000 rpm for 10 min at 4 °C. Subsequently, the separated supernatant was transferred into a clean 1.5 mL EP tube. The residue was then extracted once more with 400 μL of ethyl acetate. The combined sample extracts were evaporated at 40 °C under a nitrogen stream. 

The prepared urine sample was separated using the column/guard column combination mentioned above. The mobile phases consisted of solvent A: acetonitrile-formic acid (0.1%) and solvent B: water-formic acid (0.1%). The elution program was as follows: 0–1 min, 2% A; 1–14.5 min, 2%–15% A. The column temperature was maintained at 25 °C. The flow rate was 200 μL/min. Mass spectrometric analyses were performed on a TSQ quantum ultra-triple-quadrupole mass spectrometer. Selective ion monitoring (SIM) four ions at *m/z* 153, 167,169 and 183 for PCA, GA and their methyl metabolites respectively, in negative ion mode. Helium was used as the collision gas for collision-induced dissociation (CID). Daughter ions of the methyl metabolites were obtained under collision-induced dissociation using tandem mass spectrometry and proposed fragmentation pathways were deduced for a putative assignment of their structures.

Excretion studies of GA and PCA were performed using six rats. Each rat was orally dosed 60 mg/kg *P. capitatum* extract and housed individually in metabolic cages equipped with a urine and feces separator. After dosing, urine samples were collected at the following time intervals: before dose administration, and 0–2 h, 2–4 h, 4–6 h, 6–8 h, 8–10 h, 10–12 h, 12–24 h, and 24–48 h after dose administration. Urine samples were stored immediately at −20 °C after collection. Ten microliters of IS solution (0.1 μg/mL) was added to 100 μL urine in a 1.5 mL EP tube. Urine samples were prepared by using the same methods mentioned above and reconstituted with 100 μL of 0.1% formic acid aqueous solution, and then centrifuged at 12,000 rpm for 10 min at 4 °C. 10 μL of the reconstructed solution was loaded into UHPLC-MS/MS for GA and PCA concentration tests and excretion study.

## 4. Conclusions

In this paper, the tissue distribution and excretion studies of GA and PCA were investigated by using a UHPLC-MS/MS method. A guard column was used in this method to provide protection of the analytical column for analysis complex matrix samples. Separation programs and mass analytical parameters were also optimized to get best performance for GA and PCA analysis. After a single oral administration of *P. capitatum* extract at 60 mg/kg, GA and PCA were mainly distributed in kidney tissue. The major distribution of GA and PCA in kidney tissue helps explain the empirical folk usage of *P. capitatum* for the treatment of kidney diseases by the Miao-nationality. In the urinary excretion study, about one-sixth of the intake of GA and PCA were excreted as unchanged form, indicating that extensive metabolism of GA and PCA occurred after ingestion. Methyl metabolites of GA and PCA were detected in the urine sample and their structures were putatively identified using their characteristic fragmentation behavior. The present *in vivo* tissue distribution and excretion studies of GA and PCA after oral administration of *P. capitatum* extract in rats provide some valuable insights concerning its traditional usage for kidney diseases.

## Figures and Tables

**Table 1:** Regression equation, linear range, and LLOQ of GA and PCA in rat tissue and urine samples.

Sample	Analyte	Linearity Range (ng/mL)	Regression Equation	γ^2^	LLOQ (ng/mL)
Kidney	GA	30 ~ 3000	*Y* = 0.003733*X* + 0.017920	0.9976	30
	PCA	10 ~ 1000	*Y* = 0.015356*X* − 0.043218	0.9907	10
Lung	GA	30 ~ 3000	*Y* = 0.002384*X* − 0.031599	0.9973	30
	PCA	10 ~ 1000	*Y* = 0.015622*X* − 0.026021	0.9968	10
Heart	GA	30 ~ 3000	*Y* = 0.002114*X* − 0.003614	0.9934	30
	PCA	10 ~ 1000	*Y* = 0.009321*X* + 0.036632	0.9938	10
Liver	GA	30 ~ 3000	*Y* = 0.002397*X* − 0.042016	0.9955	30
	PCA	10 ~ 1000	*Y* = 0.011005*X* + 0.009947	0.9999	10
Spleen	GA	30 ~ 3000	*Y* = 0.006551*X* + 0.023125	0.9941	30
	PCA	10 ~ 1000	*Y* = 0.015537*X* − 0.035119	0.9953	10
Brain	GA	30 ~ 3000	*Y* = 0.006548*X* + 0.053760	0.9976	30
	PCA	10 ~ 1000	*Y* = 0.018007*X* − 0.039536	0.9904	10
Urine	GA	30 ~ 6000	*Y* = 0.007068*X* − 0.021609	0.9925	30
	PCA	10 ~ 2000	*Y* = 0.016433*X* + 0.235975	0.9907	10

**Table 2:** GA and PCA in rat urine from 0 h to 48 h after oral administration 60 mg/kg of *P. capitatum* extract (*n* = 6).

Time Interval	GA Urinary Excretion (μg)	PCA Urinary Excretion (μg)
0–2 h	199.56 ± 49.89	15.92 ± 3.98
2–4 h	90.96 ± 22.74	10.48 ± 2.62
4–6 h	46.64 ± 11.66	1.08 ± 0.27
6–8 h	13.16 ± 3.29	0.64 ± 0.16
8–10 h	6.00 ± 1.50	1.04 ± 0.26
10–12 h	1.52 ± 0.38	0.39 ± 0.39
12–24 h	3.60 ± 0.90	ND
24–48 h	ND	ND
Total	361.44 ± 90.69	29.55 ± 7.68
Excretion rate	14.60% ± 3.66%	15.72% ± 4.09%

ND, not detectable.

## Supplementary Materials

Supplementary materials can be accessed at: http://www.mdpi.com/1420-3049/21/4/399/s1.

## References

[1] S.O.P. Commission (2010). Pharmacopoeia of the People's Republic of China.

[2] Ma F., Zhao Y., Gong X., Xie Y., Zhou X. (2014). Optimization of quercitrin and total flavonoids extraction from Herba Polygoni Capitati by response surface methodology. Pharmacogn. Mag..

[3] Li X., Yu M., Meng D., Li Z., Zhang L. (2007). A new chromone glycoside from *Polygonum capitatum*. Fitoterapia.

[4] Yu M., Li Z.-L., Li N., Li X. (2008). Chemical constituents of the aerial parts of *Polygonum capitatum*. J. Shenyang Pharm. Univ..

[5] Yongjun L., Hongfeng L., Yonglin W. (2000). Studies on the chemical constituents of flavonoids from *Polygonum capitatum*. Chin. Pharm. J..

[6] Yan X.-L., Li C.-Q., Liu Y.-X., Chang X., Kang W.-Y. (2010). Antioxidant Activity of *Polygonum capitatum*. China Pharm..

[7] Wang P.-Q., Zhang X.-N., Liu Y.-X., Li C.-Q., Kang W.-Y. (2013). Antibacterial Activities of Nine Polygonaceae Plants. Chin. J. Exp. Tradit. Med. Form..

[8] Liao S.-G., Zhang L.-J., Sun F., Zhang J.-J., Chen A.Y., Lan Y.-Y., Li Y.-J., Wang A.-M., He X., Xiong Y. (2011). Antibacterial and anti-inflammatory effects of extracts and fractions from *Polygonum capitatum*. J. Ethnopharmacol..

[9] Roy B., Das A., Bhaumik U., Sarkar A.K., Bose A., Mukharjee J., Chakrabarty U.S., Das A.K., Pal T.K. (2010). Determination of gemifloxacin in different tissues of rat after oral dosing of gemifloxacin mesylate by LC-MS/MS and its application in drug tissue distribution study. J. Pharm. Biomed. Anal..

[10] Li Q., Xie L., Zhang J., Weina P.J. (2008). The distribution pattern of intravenous [^14^C] artesunate in rat tissues by quantitative whole-body autoradiography and tissue dissection techniques. J. Pharm. Biomed. Anal..

[11] Bent S., Ko R. (2004). Commonly used herbal medicines in the United States: A review. Am. J. Med..

[12] Lu T., Yang J., Gao X., Chen P., Du F., Sun Y., Wang F., Xu F., Shang H., Huang Y. (2008). Plasma and urinary tanshinol from *Salvia miltiorrhiza* (Danshen) can be used as pharmacokinetic markers for cardiotonic pills, a cardiovascular herbal medicine. Drug Metab. Dispos..

[13] Lazarowych N.J., Pekos P. (1998). Use of fingerprinting and marker compounds for identification and standardization of botanical drugs: Strategies for applying pharmaceutical HPLC analysis to herbal products. Drug Inf. J..

[14] Puchert T., Lochmann D., Menezes J.C., Reich G. (2010). Near-infrared chemical imaging (NIR-CI) for counterfeit drug identification—A four-stage concept with a novel approach of data processing (Linear Image Signature). J. Pharm. Biomed. Anal..

[15] Sroka Z., Cisowski W. (2003). Hydrogen peroxide scavenging, antioxidant and anti-radical activity of some phenolic acids. Food Chem. Toxicol..

[16] Sheng Y.-X., Li L., Wang Q., Guo H.-Z., Guo D.-A. (2005). Simultaneous determination of gallic acid, albiflorin, paeoniflorin, ferulic acid and benzoic acid in Si–Wu decoction by high-performance liquid chromatography DAD method. J. Pharm. Biomed. Anal..

[17] Tseng T.-H., Hsu J.-D., Lo M.-H., Chu C.-Y., Chou F.-P., Huang C.-L., Wang C.-J. (1998). Inhibitory effect of Hibiscus protocatechuic acid on tumor promotion in mouse skin. Cancer Lett..

[18] Aruoma O.I., Murcia A., Butler J., Halliwell B. (1993). Evaluation of the antioxidant and prooxidant actions of gallic acid and its derivatives. J. Agric. Food Chem..

[19] Wang H., Provan G.J., Helliwell K. (2003). Determination of hamamelitannin, catechins and gallic acid in witch hazel bark, twig and leaf by HPLC. J. Pharm. And Biomed. Anal..

[20] Tseng T.-H., Wang C.-J., Kao E.-S., Chu H.-Y. (1996). Hibiscus protocatechuic acid protects against oxidative damage induced by *tert*-butylhydroperoxide in rat primary hepatocytes. Chem. Biol. Int..

[21] Liu C.-L., Wang J.-M., Chu C.-Y., Cheng M.-T., Tseng T.-H. (2002). *In vivo* protective effect of protocatechuic acid on *tert*-butyl hydroperoxide-induced rat hepatotoxicity. Food Chem. Toxicol..

[22] Lu Z., Nie G., Belton P.S., Tang H., Zhao B. (2006). Structure–activity relationship analysis of antioxidant ability and neuroprotective effect of gallic acid derivatives. Neurochem. Int..

[23] Peng C.-C., Hsieh C.-L., Wang H.-E., Chung J.-Y., Chen K.-C., Peng R.Y. (2012). Ferulic acid is nephrodamaging while gallic acid is renal protective in long term treatment of chronic kidney disease. Clin. Nutr..

[24] Shi X., Xiao C., Wang Y., Tang H. (2012). Gallic acid intake induces alterations to systems metabolism in rats. J. Proteome Res..

[25] Margalef M., Pons Z., Bravo F.I., Muguerza B., Arola-Arnal A. (2015). Tissue distribution of rat flavanol metabolites at different doses. J. Nutr. Biochem..

[26] Dok-Go H., Lee K.H., Kim H.J., Lee E.H., Lee J., Song Y.S., Lee Y.-H., Jin C., Lee Y.S., Cho J. (2003). Neuroprotective effects of antioxidative flavonoids, quercetin,(+)-dihydroquercetin and quercetin 3-methyl ether, isolated from *Opuntia ficus-indica* var. Saboten. Brain Res..

[27] Manach C., Donovan J.L. (2004). Pharmacokinetics and metabolism of dietary flavonoids in humans. Free Radic. Res..

[28] Del Rio D., Rodriguez-Mateos A., Spencer J.P. E., Tognolini M., Borges G., Crozier A. (2013). Dietary (poly) phenolics in human health: Structures, bioavailability, and evidence of protective effects against chronic diseases. Antioxid. Redox Signal..

[29] Manach C., Scalbert A., Morand C., Rémésy C., Jiménez L. (2004). Polyphenols: Food sources and bioavailability. Am. J. Clin. Nutr..

[30] Masucci J.A., Mahan A.D., Kwasnoski J.D., Caldwell G.W. (2012). A Novel Method for Determination of Drug Distribution in Rat Brain Tissue Sections by LC/MS/MS: Functional Tissue Microanalysis. Curr. Top. Med. Chem..

[31] Liu D.Q., Hop C.E.C.A. (2005). Strategies for characterization of drug metabolites using liquid chromatography-tandem mass spectrometry in conjunction with chemical derivatization and on-line H/D exchange approaches. J. Pharm. Biomed. Anal..

[32] Cooks R.G., Ouyang Z., Takats Z., Wiseman J.M. (2006). Ambient Mass Spectrometry. Science.

[33] Volpi N., Bergonzini G. (2006). Analysis of flavonoids from propolis by on-line HPLC-electrospray mass spectrometry. J. Pharm. Biomed. Anal..

[34] Ma F., Gong X., Zhou X., Zhao Y., Li M. (2015). An UHPLC-MS/MS method for simultaneous quantification of gallic acid and protocatechuic acid in rat plasma after oral administration of *Polygonum capitatum* extract and its application to pharmacokinetics. J. Ethnopharmacol..

[35] Kim S.-H., Jun C.-D., Suk K., Choi B.-J., Lim H., Park S., Lee S.H., Shin H.-Y., Kim D.-K., Shin T.-Y. (2006). Gallic acid inhibits histamine release and pro-inflammatory cytokine production in mast cells. Toxicol. Sci..

[36] Soleas G.J., Grass L., Josephy P.D., Goldberg D.M., Diamandis E.P. (2002). A comparison of the anticarcinogenic properties of four red wine polyphenols. Clin. Biochem..

[37] Jang A., Srinivasan P., Lee N.Y., Song H.P., Lee J.W., Lee M., Jo C. (2008). Comparison of hypolipidemic activity of synthetic gallic acid–linoleic acid ester with mixture of gallic acid and linoleic acid, gallic acid, and linoleic acid on high-fat diet induced obesity in C57BL/6 Cr Slc mice. Chem. Biol. Interact..

[38] Hsu C.-L., Yen G.-C. (2007). Effect of gallic acid on high fat diet-induced dyslipidaemia, hepatosteatosis and oxidative stress in rats. Br. J. Nutr..

[39] Priscilla D.H., Prince P.S. M. (2009). Cardioprotective effect of gallic acid on cardiac troponin-T, cardiac marker enzymes, lipid peroxidation products and antioxidants in experimentally induced myocardial infarction in Wistar rats. Chem. Biol. Interact..

[40] Sabu M.C., Kuttan R. (2002). Anti-diabetic activity of medicinal plants and its relationship with their antioxidant property. J. Ethnopharmacol..

[41] Shahrzad S., Bitsch I. (1998). Determination of gallic acid and its metabolites in human plasma and urine by high-performance liquid chromatography. J. Chromatogr. B: Biomed. Sci. Appl..

[42] Booth A.N., Masri M.S., Robbins D.J., Emerson O.H., Jones F.T., DeEds F. (1959). The metabolic fate of gallic acid and related compounds. J. Biol. Chem..

[43] Shahrzad S., Aoyagi K., Winter A., Koyama A., Bitsch I. (2001). Pharmacokinetics of gallic acid and its relative bioavailability from tea in healthy humans. J. Nutr..

[44] Hodgson J.M., Morton L.W., Puddey I.B., Beilin L.J., Croft K.D. (2000). Gallic acid metabolites are markers of black tea intake in humans. J. Agric. Food Chem..

[45] Zong L., Inoue M., Nose M., Kojima K., Sakaguchi N., Isuzugawa K., Takeda T., Ogihara Y. (1999). Metabolic fate of gallic acid orally administered to rats. Biol. Pharm. Bull..

