# Supplementary material for: The Tissue Distribution and Urinary Excretion Study of Gallic Acid and Protocatechuic Acid after Oral Administration of Polygonum Capitatum Extract in Rats
Source: Molecules. 2016 Mar 24;21(4):399. doi: 10.3390/molecules21040399 (PMC6273519; doi:10.3390/molecules21040399)
Supplement: Supplementary file 1 [file molecules-21-00399-s001.pdf]

Supplementary Materials: The Tissue Distribution and Urinary Excretion Study of Gallic Acid and Protocatechuic Acid after Oral Administration of *Polygonum Capitatum* Extract In Rats

Feng-Wei Ma, Qing-Fang, Xin Zhou, Xiao-Jian Gong, Yang Zhao, Hua-Guo Chen and Chao Zhao

Table S1. Summary of accuracy, precision, and extract recovery of GA and PCA in rat kidney and urine (n = 6).

Tissue	Analyte	Nominal Concentration (ng/mL)	Intra-day			Inter-day			Extract Recovery (%)
			Measured Concentration (ng/mL)	Precision (% RSD)	Accuracy (% RE [a])	Measured Concentration (ng/mL)	Precision (% RSD)	Accuracy (% RE [a])	
Kidney	GA	30	32.43	16.31	8.10	33.14	10.53	10.47	81.51 ± 5.16
		300	345.34	6.53	15.11	307.36	6.24	2.45	80.26 ± 4.87
		3000	3112.67	5.68	3.76	2808.94	3.89	−6.37	84.94 ± 6.29
	PCA	10	9.45	14.77	−5.50	9.53	10.35	−4.70	81.47 ± 5.19
		100	111.56	6.42	11.56	106.34	6.98	6.34	81.96 ± 7.46
		1000	889.21	4.98	−11.08	888.34	8.93	−11.17	86.45 ± 6.51
Lung	GA	30	31.94	13.79	6.47	27.87	14.22	−7.10	82.02 ± 5.27
		300	289.11	4.69	−3.63	307.63	8.76	2.54	82.44 ± 4.96
		3000	2788.56	9.71	−7.05	3117.82	4.98	3.93	84.21 ± 7.30
	PCA	10	11.37	14.21	13.70	10.15	11.34	1.50	86.37 ± 6.91
		100	89.74	8.85	−0.26	88.58	5.73	−1.42	86.56 ± 8.64
		1000	1137.16	5.98	13.72	897.16	7.52	−0.28	90.87 ± 7.63
Heart	GA	30	26.67	9.81	−11.10	31.09	10.43	3.63	81.06 ± 6.15
		300	277.75	7.94	−7.42	311.32	7.99	3.77	80.68 ± 7.84
		3000	2805.48	9.28	−6.48	2904.89	8.98	−3.17	84.99 ± 9.26
	PCA	10	9.17	10.34	−8.30	8.67	12.09	−3.30	86.42 ± 9.34
		100	104.22	9.70	4.22	87.34	9.32	−2.66	82.46 ± 7.59
		1000	958.09	9.43	−4.19	1122.36	8.63	12.24	86.93 ± 6.63
Liver	GA	30	30.96	13.47	3.20	30.37	10.23	1.23	85.49 ± 6.27
		300	307.35	9.55	2.45	299.05	9.78	−0.32	84.48 ± 5.97
		3000	3227.71	10.63	7.59	2943.88	8.13	−1.87	80.67 ± 7.31
	PCA	10	8.92	11.22	−0.80	8.71	14.10	−1.29	82.93 ± 6.91
		100	90.81	7.89	−9.19	101.38	10.12	1.38	81.50 ± 8.57
		1000	1068.5	8.45	6.85	1068.17	8.11	6.82	86.68 ± 7.62

Table S1. *Cont*.

Spleen	GA	30	27.35	11.32	−8.83	31.43	7.74	4.77	81.14 ± 6.38
		300	309.87	8.78	3.29	313.12	7.43	4.37	87.36 ± 9.65
		3000	2852.75	9.58	−4.91	3123.47	6.61	4.12	80.96 ± 8.42
	PCA	10	10.26	9.98	2.60	10.38	10.21	3.80	83.75 ± 7.14
		100	93.70	10.01	−6.30	95.12	7.36	−4.88	81.91 ± 6.73
		1000	989.24	5.45	−1.08	1066.98	6.12	6.70	86.93 ± 8.47
Brain	GA	30	29.08	9.33	−3.07	28.49	7.49	−5.03	85.56 ± 7.26
		300	284.45	8.59	−5.18	297.59	9.72	−0.80	90.25 ± 8.85
		3000	3124.53	8.66	4.16	3056.57	10.39	1.89	80.71 ± 9.74
	PCA	10	9.57	12.09	−4.30	9.48	9.97	−5.20	91.72 ± 8.21
		100	98.86	8.78	−1.14	93.64	7.99	−6.56	81.96 ± 5.79
		1000	1006.50	8.23	0.65	1011.42	5.44	1.14	87.41 ± 6.54
Urine	GA	60	64.84	4.71	8.07	64.80	9.97	8.00	90.10 ± 3.75
		300	278.01	10.90	−7.33	284.43	5.26	−5.19	86.49 ± 8.48
		3000	2707.50	2.15	−9.75	2740.56	4.32	−8.65	86.84 ± 8.43
	PCA	20	18.10	10.56	−9.50	19.46	12.18	−2.70	84.64 ± 5.08
		100	96.09	7.07	−3.91	96.09	3.65	−3.91	90.43 ± 5.38
		1000	892.25	1.69	−10.78	885.49	1.48	−11.45	90.86 ± 7.15

[a] RE is expressed as (measured concentration/nominal concentration) × 100%.

Table S2. Stability data of GA and PCA in rat tissues and urine under different conditions.

Status	Analyte	Nominal Concentration (ng/mL)	Room Temperature Stability			Post-preparative Stability		
			Found Concentration (Mean ± SD; ng/mL)	Accuracy (%)	RSD (%)	Found Concentration (Mean ± SD; ng/mL)	Accuracy (%)	RSD (%)
**Kidney**	GA	1700	1767.45 ± 430.91	103.97	8.43	1568.35 ± 91.44	92.26	9.54
	PCA	85	84.51 ± 23.03	99.42	3.62	81.98 ± 13.45	96.45	6.34
**Lung**	GA	1700	1553.68 ± 487.37	91.39	11.41	1568.35 ± 379.42	92.26	9.26
	PCA	85	82.19 ± 39.42	96.69	10.60	83.16 ± 26.13	97.84	8.97
**Heart**	GA	1700	1642.39 ± 134.16	96.61	8.36	1624.27 ± 114.22	95.55	6.69
	PCA	85	87.73 ± 42.39	103.21	8.36	83.87 ± 24.38	98.67	10.21
**Liver**	GA	1700	1652.30 ± 256.03	97.19	5.41	1528.42 ± 258.34	89.91	7.19
	PCA	85	85.09 ± 9.09	100.11	3.98	86.2 ± 42.28	101.41	10.11
**Spleen**	GA	1700	1675.32 ± 346.67	98.55	7.24	1545.75 ± 34.44	90.93	8.55
	PCA	85	84.11 ± 32.75	98.95	8.16	80.33 ± 17.31	94.51	9.33
**Brain**	GA	1700	1680.08 ± 163.32	98.33	6.83	1452.94 ± 169.45	85.47	7.37
	PCA	85	83.11 ± 22.37	97.78	8.91	82.82 ± 10.33	97.44	9.42
**Urine**	GA	1700	1653.68 ± 299.29	97.28	4.28	1663.38 ± 123.76	97.85	5.39
	PCA	85	83.91 ± 23.68	98.72	10.62	84.79 ± 8.57	99.75	11.72
**Kidney**	GA	1700	1546.98 ± 190.34	91.00	10.22	1469.98 ±187.78	86.47	8.33
	PCA	85	78.92 ± 12.14	92.85	7.34	73.49 ± 34.57	86.46	6.39
**Lung**	GA	1700	1497.26 ± 386.36	88.07	10.53	1507.41 ± 268.31	88.67	6.92
	PCA	85	75.58 ± 28.33	91.35	6.94	74.71 ± 13.26	87.89	9.88
**Heart**	GA	1700	1392.94 ± 416.13	81.94	6.74	1532.94 ± 224.98	90.17	7.78
	PCA	85	75.58 ± 31.28	88.92	7.25	73.53 ± 27.13	86.51	11.12
**Liver**	GA	1700	1548.24 ± 145.03	91.07	6.52	1419.76 ± 247.39	83.52	8.21
	PCA	85	79.26 ± 19.87	93.25	8.49	72.47 ± 31.82	85.26	9.20
**Spleen**	GA	1700	1421.88 ± 235.76	83.64	5.47	1470.63 ± 444.21	86.51	9.56
	PCA	85	75.85 ± 22.67	89.24	6.29	75.29 ± 28.13	88.58	10.44
**Brain**	GA	1700	1467.06 ± 232.63	86.30	9.74	1489.12 ± 245.69	87.60	8.48
	PCA	85	77.43 ± 31.17	91.09	7.86	74.12 ± 33.05	87.20	8.53
**Urine**	GA	1700	1558.21 ± 145.16	91.66	9.28	1484.42±139.77	87.32	12.46
	PCA	85	79.88 ±16.88	93.98	11.15	75.95 ± 6.43	89.35	7.53

Table S3. Tissue contents of GA and PCA in various rat organs after oral administrations 60 mg/kg of *P. capitatum* extract (n = 6).

**Tissue**	**Analytes**	**Concentration (ng/g)**						**Mean ± SD**
		**1**	**2**	**3**	**4**	**5**	**6**	
Kidney	GA	1348.09	1245.47	1930.40	1121.44	785.68	880.65	1218.62 ± 408.71
	PCA	46.93	55.27	63.18	38.88	30.39	29.22	43.98 ± 13.66
Lung	GA	382.01	360.02	279.16	160.23	176.66	190.42	258.08 ± 96.93
	PCA	13.59	22.23	20.18	13.80	24.51	22.57	19.48 ± 4.68
Heart	GA	133.09	163.06	285.01	202.85	269.41	238.91	215.39 ± 59.98
	PCA	ND	ND	ND	ND	ND	ND	ND
Liver	GA	186.98	199.98	235.49	269.43	287.83	209.61	231.55 ± 40.21
	PCA	12.81	19.11	26.27	36.73	17.96	7.94	20.14 ± 10.21
Spleen	GA	ND	5.86	22.03	ND	ND	11.54	13.14 ± 8.20
	PCA	ND	ND	ND	ND	ND	ND	ND
Brain	GA	ND	ND	ND	ND	ND	ND	ND
	PCA	ND	ND	ND	ND	ND	ND	ND

ND, not detectable.

Table S4. Values of tube lens offset (V), collision pressure (m Torr) and collision energy (eV) for the parent ions-product ions transitions.

**Analytes**	**Transition**	**Tube Lens Offset (V)**	**Collision Pressure (m Torr)**	**Collision Energy (eV)**
GA	169.012→125.042	73	1.8	15
PCA	153.114→109.201	77	1.8	15
IS	326.930→192.017	102	1.8	26

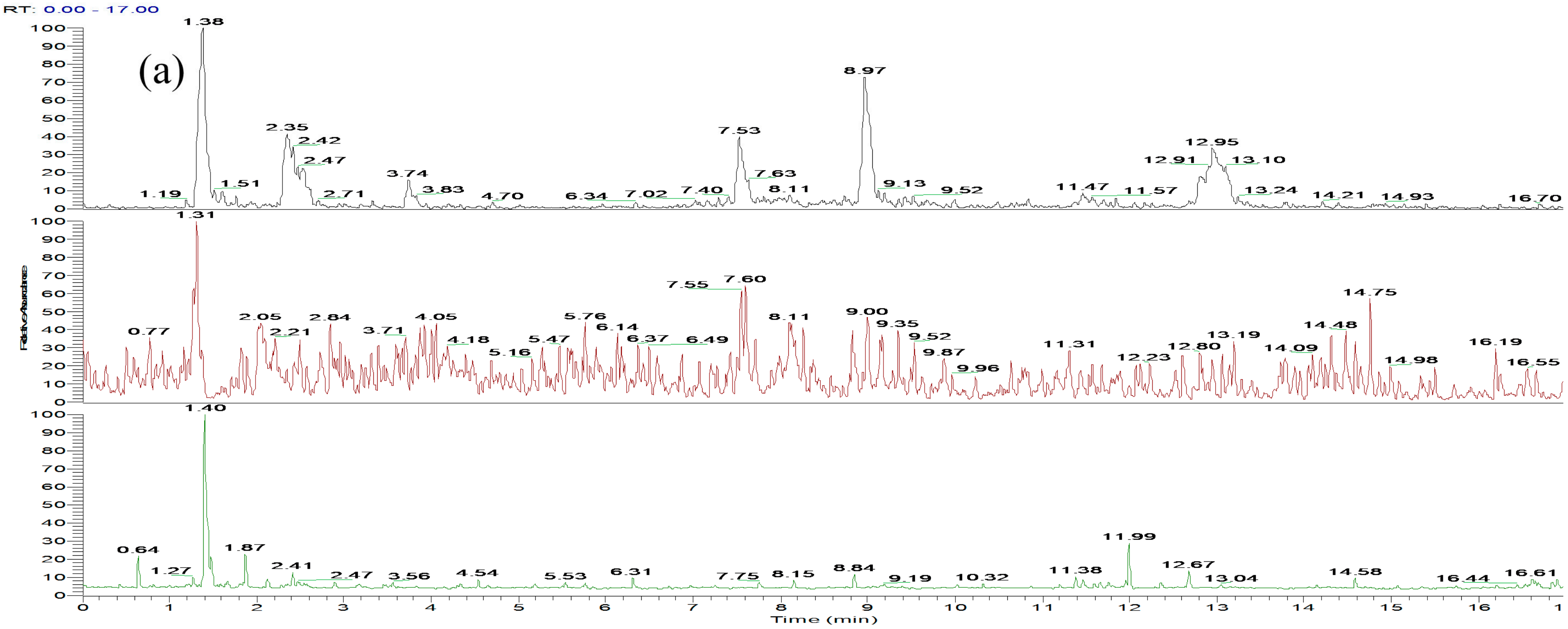

Figure S1. *Cont.*

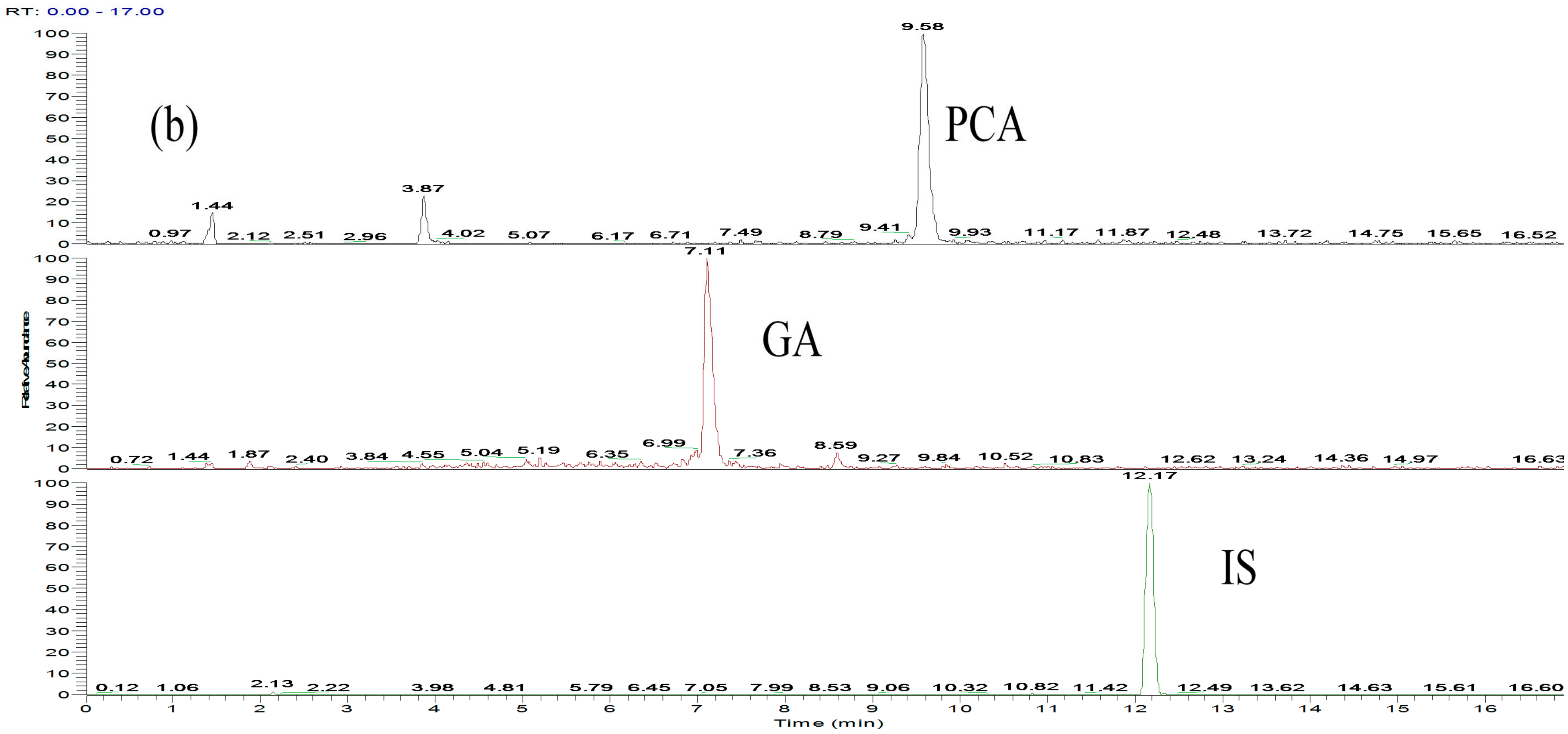

Figure S1. *Cont.*

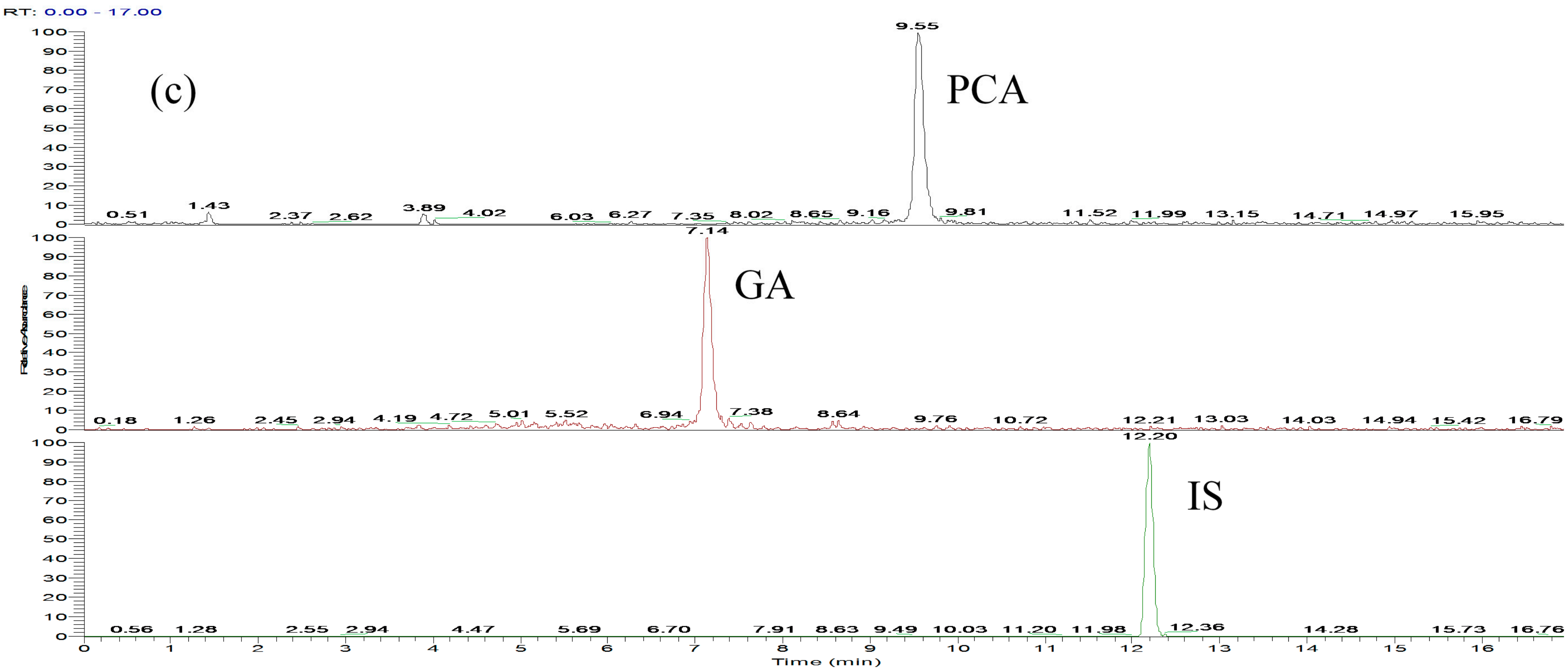

Figure S1. Representative MRM chromatograms of GA, PCA and IS (bergenin) in rat urine: (**a**) blank urine; (**b**) blank urine spiked with GA, PCA and IS at concentration of 150.02, 49.75, and 10.01 ng/mL, respectively; (**c**) urine at 0–2 h after oral administration of 60 mg/kg *Polygonum capitatum* extract.

(a)

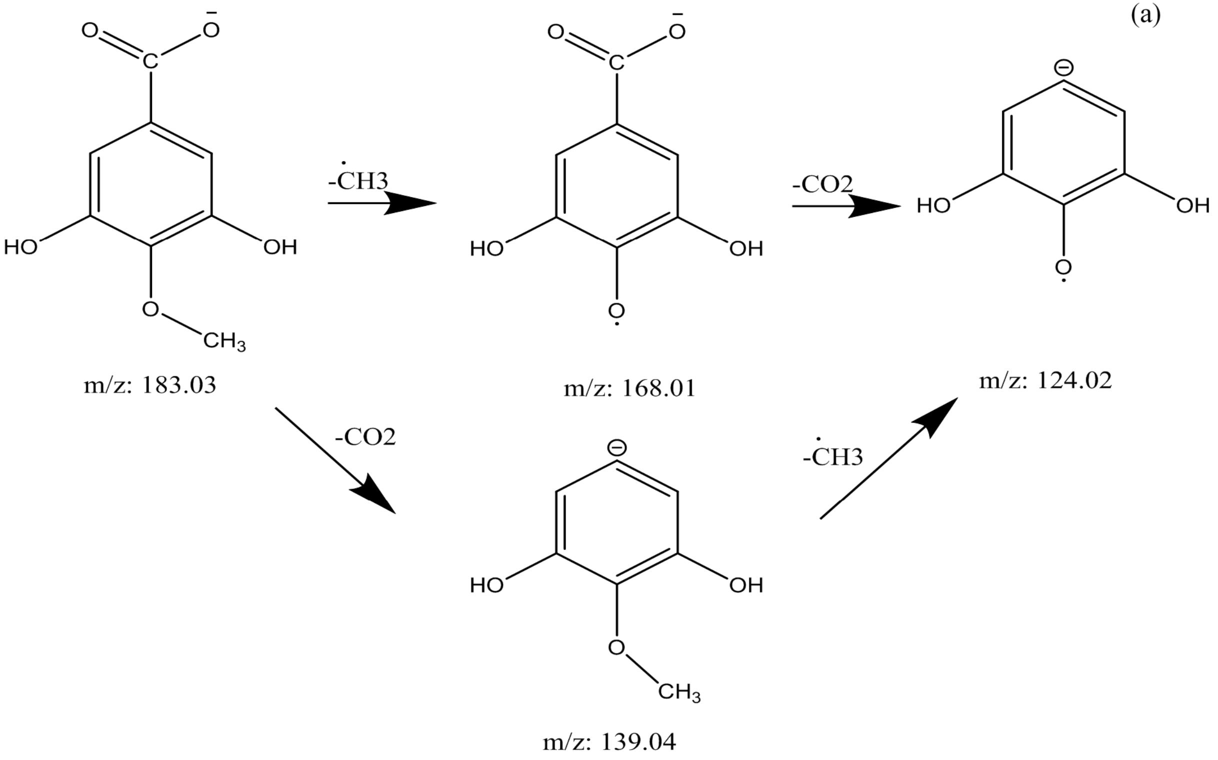

Figure S2. *Cont.*

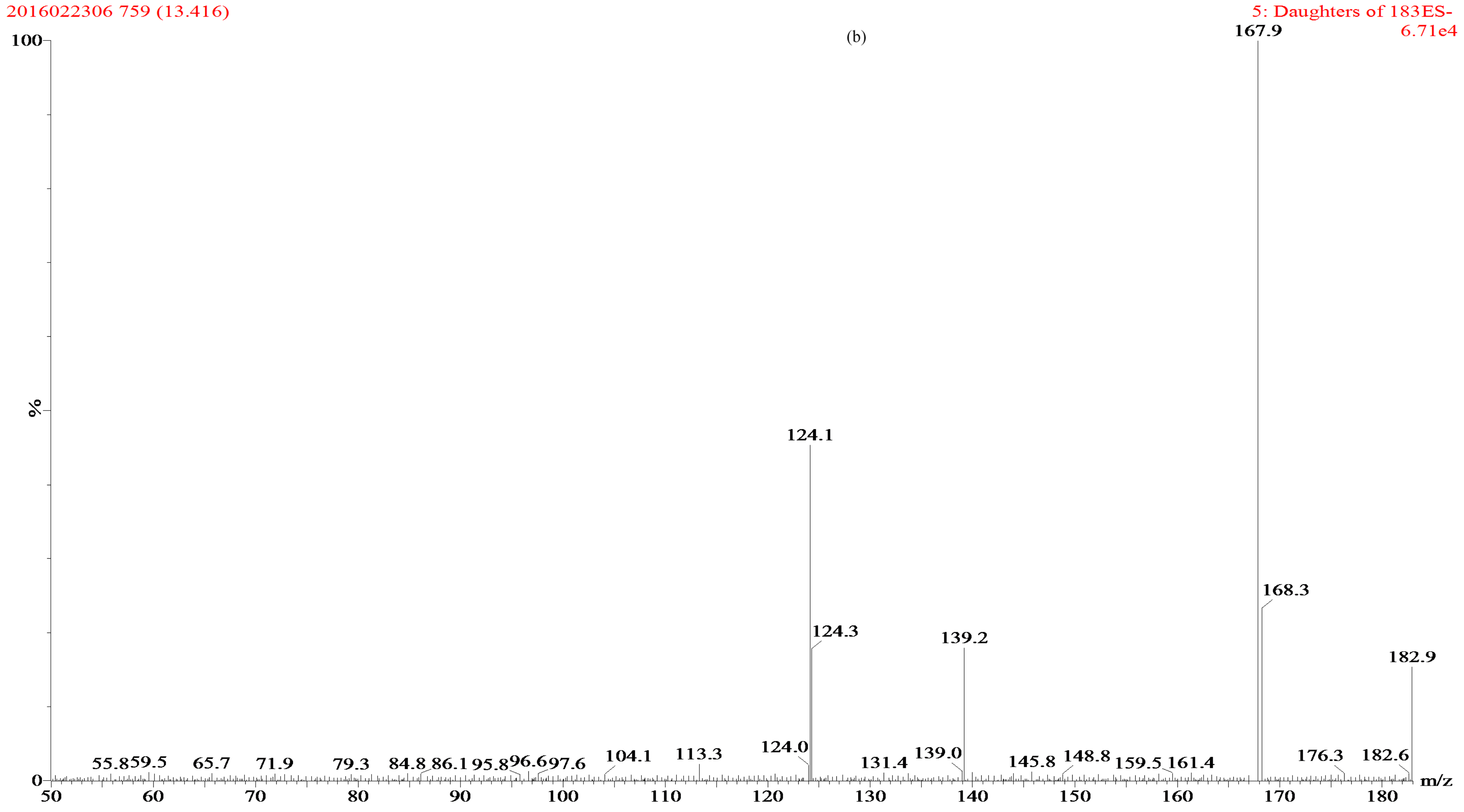

Figure S2. (**a**) Proposed fragmentation patterns of 4-OMeGA; and (**b**) its MS/MS spectra under collision energy of 15 eV.
